# Prevalence of post-traumatic stress disorder during the COVID-19 pandemic in children: a review and suggested solutions

**DOI:** 10.1186/s43045-022-00240-x

**Published:** 2022-09-27

**Authors:** Zahra Karbasi, Parisa Eslami

**Affiliations:** 1 Department of Health Information Sciences, Faculty of Management and Medical Information Sciences, Kerman University of Medical Sciences, Kerman, Iran; 2 Department of Health Information Management, School of Allied Medical Sciences, Tehran University of Medical Sciences, Tehran, Iran

**Keywords:** Children, COVID-19, Post-traumatic stress disorder

## Abstract

**Background:**

The outbreak of coronavirus began in China in December 2019. It became a pandemic and a public health emergency. There have been numerous reports related to post-traumatic stress disorder outbreaks in the COVID-19 crisis. After a natural disaster, children are at a higher risk for post-traumatic stress disorder. The current study is a review of the scientific literature on the effect of COVID-19 on the prevalence of symptoms of post-traumatic stress disorder in children.

We searched PubMed, Web of Science, and Scopus databases until February 02, 2022. The search strategy was based on a combination of the following keywords “child,” “COVID-19,” and “post-traumatic stress disorder.”

**Results:**

By searching the Web of Science, Scopus, and PubMed databases, 173 articles were retrieved. After reviewing the inclusion criteria and in terms of eligibility, 10 articles met the inclusion criteria out of the remaining 46 articles. Based on the findings, 80% of the articles were cross-sectional and 20% of them were longitudinal. The articles reviewed in this study reported an increase in the prevalence of post-traumatic stress disorder in children during or after the COVID-19 pandemic.

**Conclusions:**

In summary, the findings of this review showed that restrictions and fears of COVID-19 had negative psychological effects on children. As well, one of the most important issues that arose at the time of the tragedy was that children were suffering from post-traumatic stress disorder. Given that post-traumatic stress disorder can be treated, it is essential to choose the appropriate therapeutic intervention approach in order to better deal with the negative effects in children.

## Background

The outbreak of coronavirus disease 2019 (COVID-19) began in China in December, 2019 [1], which rapidly spread all around the world [2]. Eventually, it became a pandemic and a public health emergency [3]. The crisis caused stress among the public, and the World Health Organization (WHO) expressed concern about the mental health of people during the pandemic as well as its psychological and social consequences [4]. Restrictive and quarantine measures affected people’s lives, especially people’s mental health status, and psychological problems such as depression, stress, and anxiety are expected to increase as consequences [2]. Research results showed that anxiety disorders, depression, and post-traumatic stress disorder (PTSD) usually increase after major crises [5–7]. Evidence suggests that the consequences of the coronavirus pandemic are associated with PTSD, psychological distress, psychiatric disorders, pathological and psychological symptoms, and stress [8–10]. Psychiatric disorders and PTSD place a significant burden on individuals [11]. PTSD is a type of psychiatric condition caused either by a crisis or by traumatic factors [12, 13]. This psychiatric disorder caused by critical events such as natural disasters, severe injuries, death, and threats directly or indirectly affect people [14]. The prevalence rate of PTSD in the COVID-19 pandemic varies among different populations. For example, 29.5% of PTSD symptoms were found in the Italian population [15]. The prevalence rate of this disorder in young adults in the USA is reported to be 31.8% [16]. A recent study performed in China estimated the prevalence rates of PTSD and depression among students by passing 1 month from COVID-19 at 2.7 and 9.0%, respectively [17].

It was demonstrated that some age groups are more vulnerable to the psychological consequences of this pandemic. Due to the fact that children are in a critical period of their development, they need special care for their mental health [18]. As well, they can express their stress in different ways [19]. Stress, anxiety, and PTSD were shown to have negative and debilitating effects on a child’s performance [20]. After a natural disaster, children are at a higher risk for PTSD [21]. Children may experience various consequences like PTSD in the context of pandemics [22]. According to a study by Saurabh and Ranjan, 68% of children quarantined during the COVID-19 pandemic suffered from mental illnesses [23]. A recent review found that the COVID-19 pandemic has a significant impact on mental health and more negative consequences like PTSD among vulnerable age groups, including children [24]. Based on the research, the prevalence of symptoms of PTSD has expanded due to the prevalence of COVID-19. Accordingly, the prevalence rate of PTSD among people aged between 14 and 35 in China is reported to be 12.8% [25].

Numerous articles [26–28] have previously reviewed the effects of COVID-19 and the prevalence of PTSD symptoms among different groups and individuals. The current study is a review of the scientific literature on the effect of COVID-19 on the prevalence of symptoms of PTSD in children.

## Methods

### Search strategy

**Fig. 1:**
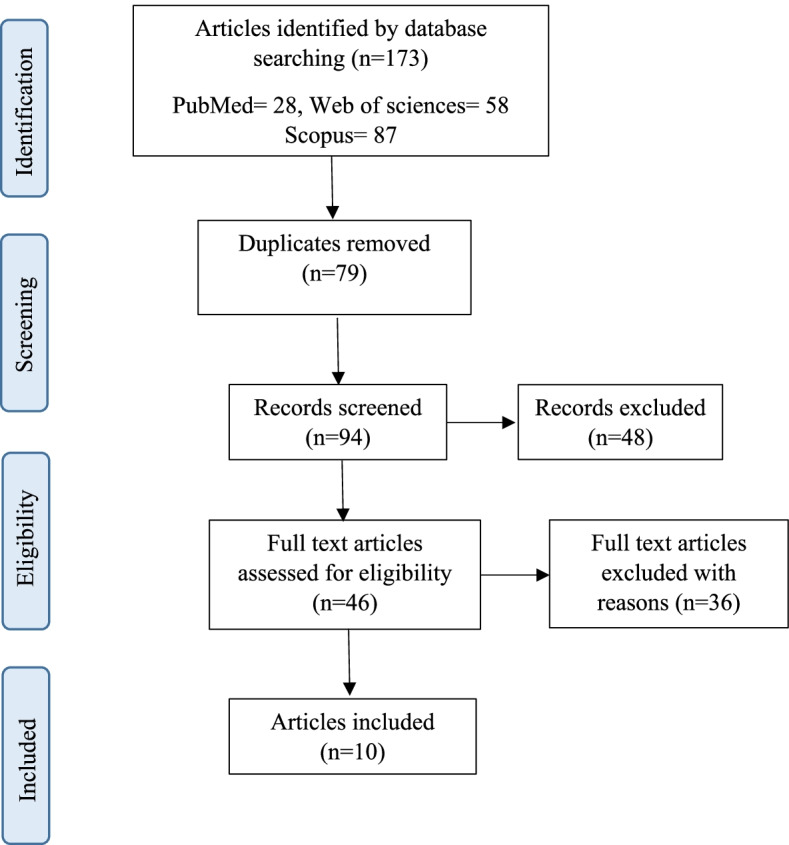
The PRISMA diagram of the study selection process

This review was performed in terms of the Preferred Reporting Items for Systematic Reviews and Meta-analyses (PRISMA) checklist [29]. We searched PubMed, Web of Science, and Scopus databases until February 02, 2022. The search strategy was based on a combination of the following keywords “child,” “COVID-19,” and “post-traumatic stress disorder.” Boolean search strategies are based on these keywords tailored to each database. PRISMA flow chart of the article selection process is presented in Fig. 1.

### Inclusion criteria

Articles were included in the study if they met the following inclusion criteria:

- Articles published in the English language
- Articles focusing on the effect of COVID-19 on the prevalence of PTSD in children
- Having accordance with the search strategy

### Exclusion criteria

- Review or systematic review, books, letters to editors, short communication, reports, and commentaries
- Articles whose abstracts and full text were not available

### Screening and article selection phase

In this phase, the retrieved articles were entered into the EndNote software, and duplicates were removed. Thereafter, two independent reviewers reviewed the titles and abstracts of the articles to identify relevant articles. Disagreements were mentioned and resolved through consensus in a joint meeting. Finally, the related articles were downloaded and then evaluated for eligibility and data extraction.

### Analysis

The data extraction form of the included articles was designed by the authors in Excel, and the following information was extracted for each article: author, year, country, type of study, objective, participants, age, measures, and the main findings. The obtained data were presented with descriptive statistics and percentages. The general characteristics of the included studies are summarized in Table 1.

## Results

### Study selection

At this stage, by searching the Web of Science, Scopus, and PubMed databases, 173 articles were retrieved. Of these, 79 duplicate articles were removed and 94 articles entered the screening process. We excluded 48 articles, and after reviewing the inclusion criteria and in terms of eligibility, 10 articles met the inclusion criteria out of the remaining 46 articles.

#### Characteristics of articles

A summary of general specifications for the included studies is provided in Table 1.

**Table 1:** Main characteristics of the included studies

Author	Year	Country	Type of study	Objective	Participants	Age, years (mean±SD)	Measures	Main findings
Chen et al. [30]	2021	China	Cross-sectional	To evaluate the prevalence of PTSD symptoms in students in Wuhan	Children	12.26 ± 2.14 years	Online questionnaire	According to the findings, 11.5% of students had clinical criteria related to PTSD symptoms.
Davico et al. [31]	2021	Italy	Cross-sectional	Assessing the mental impact of COVID-19 on adults and children	Adult and children	12.3 ± 3.2 years	Online questionnaire	The results showed that 30.9% of children were at high risk for PTSD in the COVID-19 crisis.
Eray et al. [32]	2021	Turkey	Cross**-**sectional	Evaluation of admission of children with psychiatric symptoms during and before the COVID-19 pandemic	Children	First group: 14.4±2.53 Second group: 14.2±3.05	Patients data	The results showed an increase in diagnoses of PTSD among children during the COVID-19 pandemic.
He et al. [33]	2021	China	Cross-sectional	To assess children’s psychological health conditions in the COVID-19 pandemic	Children	11.83±0.79 years	Online questionnaire	The results showed that children’s rates of PTSD were low (the prevalence of PTSD was 2.04%)
Ma et al. [34]	2021	China	Cross-sectional	To assess the impact of the COVID-19 pandemic on psychological health among Chinese children	Parents and children	7–15 years	Online questionnaire	The results showed that the COVID-19 pandemic has caused PTSD, as well as 20.7 and 7.2% of children experienced PTSD.
Raffagnato et al. [35]	2021	Italy	Longitudinal study	To assess the impact of the COVID-19 pandemic on the mental health of children and adolescents with psychiatric disorders	Children and their parents	13.4±2.77 years	Semi-structured interview and questionnaires	No significant differences were found in the psychological behavior of patients, as evidenced by a significant reduction in post-traumatic stress problems.
Raymond et al. [36]	2022	Canada	Longitudinal study	To assess the effects of mental distress in children during the COVID-19 pandemic	Children	9–14 years	Online questionnaire	The results showed that PTS symptoms increased in children aged 9 to 11 years old.
Sayed et al. [37]	2021	Saudi Arabia	Cross-sectional	To assess for PTSD symptoms in children/adolescents in Saudi Arabia during the COVID-19 pandemic	Children	12.25±3.77 years	Online questionnaire	The results showed that the COVID-19 pandemic was associated with the prevalence of PTSD in children.
Xu et al. [38]	2021	China	Cross-sectional	To investigate the prevalence of PTSD in school students in China during COVID-19	Children	8–18 years	Questionnaire	The results showed that the COVID-19 crisis has led to psychological stresses on Chinese students.
Zhang et al. [39]	2021	China	Cross-sectional	To assess the psychological health status of children discharged in the COVID-19 pandemic	Children	7–18 years	Questionnaire	An increased prevalence of PTSD was observed in discharged children.

### Year of publication

Due to the start of the pandemic of COVID-19 since 2019, most of the published articles are related to the period 2021 onwards. Based on the findings, 90% of the articles were published in 2021 as well as 10% in 2022.

### Country

Five studies were performed in China, two in Italy, and the remaining in Turkey, Canada, and Saudi Arabia.

### Type of study

Based on the findings, 80% of the articles were cross-sectional and 20% of them were longitudinal.

### Participants

According to the purpose of the reviewed articles, which was assessing the prevalence of PTSD related to COVID-19, participants in the included studies were children, students, and parents.

### Age of participants

The age group of the studied children is presented in Table 1.

### Measures

The included studies have used various measures to assess the prevalence of PTSD. In six articles, an online questionnaire was used, two articles used a questionnaire, one article analyzed patients’ data, and one article used a semi-structured interview.

### Objectives and findings

The main objectives and findings of these articles are summarized in Table 1.

## Discussion

The COVID-19 pandemic has had a profound effect on people’s lives. In the meantime, children as a sensitive and vulnerable group were affected by the negative consequences of COVID-19. Children’s mental health is one of the related and effective factors in their growth and development. In this review, we aimed to provide evidence on the prevalence of PTSD in children in the COVID-19 crisis.

Quarantine at home has reduced interpersonal communication and at the same time increased people’s psychological problems. Based on this reason, people become more prone to mental problems and mental disorders [40]. Children may experience different behavioral reactions based on stressful situations [36]. The articles reviewed in this study reported an increase in the prevalence of PTSD in children during or after the COVID-19 pandemic. Similarly, the results of a meta-analysis by Rezayat et al. showed that PTSD was prevalent among children and adolescents surviving from natural disasters [41]. Therefore, psychological support and paying attention to the mental health needs of children in traumatic conditions can reduce the prevalence of PTSD and other mental disorders. The prevalence of PTSD in children after the occurrence of natural disasters depends on various factors, including the time elapsed after the disaster, research methods, and definitions of consequences and symptoms [42].

In two articles reviewed in this case, PTSD symptoms were assessed by parents. Although these studies have been shown to increase PTSD in children, parents may underestimate the degrees of distress and reaction to traumatic conditions in their children [43]. Children may not share their feelings and conversations about the event with their parents; on the other hand, it is possible for parents to understand their children without representing any symptoms. Therefore, in examining the symptoms, it is very important that the children’s own report be more prioritized [44].

### Solutions

Following the occurrence of disasters and traumatic events, children mostly experience psychological problems, especially PTSD. In this regard, awareness of appropriate treatments and interventions can greatly protect children from serious harm.

#### Psychotherapy interventions

The use of post-disaster mental health interventions is essential in this regard [45]. Counseling helps children as well as their families to recover as quickly as possible at their home or even at school. As well, therapists can follow the treatment path by performing “play therapy,” “communication with the child,” and “behavioral therapy” methods [46]. Art therapy is suitable for the treatment of children experiencing natural disasters [47]. In stressful events, the help and intervention of therapists such as pediatricians, counselors, and mental health therapists can effectively reduce children’s anxiety and fear. In addition, it is necessary to know the types of assistive methods in order to meet the mental and emotional needs of children after the disaster [48]. Helping the child to talk about his/her emotional issues and recounting problems and issues related to lost loved ones [49] will be effective in improving post-disaster problems.

In addition to the abovementioned statements, a psychosocial intervention could also help in identifying symptoms and improving children’s psychological problems. The use of group therapies for the effectiveness of treatment in children who are at risk of various types of disasters is recommended [50]. In group therapy, people with similar problems are examined in a group [51]. Several articles [52–54] have previously focused on the use of group therapy as an effective tool in the treatment of children affected by disasters. Evidence suggests that various intervention therapies in the COVID-19 pandemic, such as art therapy [55, 56] and psychosocial support services [56], can improve children’s mental health status.

#### Cognitive behavior therapy

One of the most common methods used for the treatment of PTSD is cognitive behavioral therapy (CBT), which is a combination of both cognitive and behavioral therapies [57]. CBT helps the child to control his/her anxiety and negative emotions and to master situations that cause this kind of anxiety [51]. Studies [58, 59] have shown the effectiveness of cognitive behavioral therapy for children after disasters. Findings from Lee et al.’s study showed that CBT programs can be helpful in improving the mental health status of children with autism in the COVID-19 pandemic [60].

#### Psychoeducation

One of the main components of behavioral and mental health interventions is mental education and providing the necessary training regarding disasters. Involving parents and children in these trainings will facilitate parental support and help to identify and raise awareness about disaster response [61]. It is noteworthy that psychological education for children immediately after a disaster can reduce their psychological damage and be very effective in their treatment [62].

Schools are places offering psychological education, psychological aid, and group support services and can be used to provide psychological health services or referrals to social services [63]. In the event of a disaster due to the lack of having access to a sufficient number of mental health professionals, trained teachers, or teaching staff can provide counseling services [64]. Holding psychological education seminars and activities such as play therapy, art therapy, and book therapy after an earthquake has shown that such programs have been useful for children; thus, they can talk about the problems related to the event with others [65]. Cognitive education allows children to express their feelings and to avoid feelings of being in danger [51].

#### Parental support

The most effective people in supporting children are their parents, who can influence children as agents of change. Parents can help their children to return to their normal routine of life and normal activities and keep them away from negative and bad feelings and thoughts. Parents can also protect their children from being exposed to secondary adversity [61].

#### Tele psychiatry

Tele psychiatry is used to follow patients and reduce any unnecessary travel [66], which can be used as an alternative method to face-to-face therapy, especially in the COVID-19 crisis [67].

There are several therapeutic interventions used for helping children to deal with the problems caused by experiencing disasters. By better understanding the traumatic conditions and risk factors as well as choosing the appropriate treatment strategy, interventions can be started after a natural disaster [42]. In addition to the abovementioned statement, pharmacological interventions in the treatment of pediatric PTSD based on a step-by-step approach can be effective in improving child performance [68].

#### Limitations

In this review, only the problem of PTSD was addressed, while the psychological problems caused by the COVID-19 pandemic were more prevalent among children. In addition, due to the start of the COVID-19 pandemic since 2019, the number of studies examining PTSD in children is still limited.

## Conclusions

In summary, the findings of this review showed that restrictions and fears of COVID-19 had negative psychological effects on children. As well, one of the most important issues that arose at the time of the tragedy was that children were suffering from PTSD. Given that PTSD can be treated, it is essential to choose the appropriate therapeutic intervention approach in order to better deal with the negative effects in children. In this regard, more awareness of parents as well as institutions related to child education is needed for performing effective interventions aimed at preventing and treating children’s stress. Supporting children in the COVID-19 pandemic or similar disasters using psychotherapy techniques can dramatically improve the negative and destructive thoughts of children. It is suggested that future research address more aspects of children’s mental health status in crises and natural disasters.

## Data Availability

Not applicable.

## Abbreviations

COVID-19: Coronavirus disease 2019
PTSD: Post-traumatic stress disorder
WHO: World Health Organization
